# The clinical value of proneural, classical and mesenchymal protein signatures in WHO 2021 adult-type diffuse lower-grade gliomas

**DOI:** 10.1371/journal.pone.0285732

**Published:** 2023-05-16

**Authors:** Anna Dénes, Thomas Olsson Bontell, Hanna Barchéus, Sandra Ferreyra Vega, Helena Carén, Cecilia Lindskog, Asgeir S. Jakola, Anja Smits

**Affiliations:** 1 Department of Clinical Neuroscience, Institute of Neuroscience and Physiology at the Sahlgrenska Academy, University of Gothenburg, Gothenburg, Sweden; 2 Department of Clinical Pathology, Sahlgrenska University Hospital, Gothenburg, Sweden; 3 Department of Physiology, Institute of Neuroscience and Physiology, University of Gothenburg, Sahlgrenska Academy, Gothenburg, Sweden; 4 Department of Medical Biochemistry and Cell Biology, Institute of Biomedicine, Sahlgrenska Center for Cancer Research, Sahlgrenska Academy, University of Gothenburg, Gothenburg, Sweden; 5 Department of Immunology, Genetics and Pathology, Uppsala University, Uppsala, Sweden; 6 Department of Neurosurgery, Sahlgrenska University Hospital, Gothenburg, Sweden; Instituto de Investigacion Sanitaria INCLIVA, SPAIN

## Abstract

**Objectives:**

Accumulating evidence shows that mesenchymal transition of glioblastomas is associated with a more aggressive course of disease and therapy resistance. In WHO2021-defined adult-type diffuse gliomas of lower grade (dLGG), the transition of the tumor phenotype over time, has not been studied. Most efforts to correlate proneural, classical or mesenchymal phenotype with outcome in dLGG were made prior to the WHO 2021 classification. Here, we set out to investigate if phenotype predicted survival and tumor recurrence in a clinical cohort of dLGGs, re-classified according to the 2021 WHO criteria.

**Methods:**

Using a TMA-based approach with five immunohistochemical markers (EGFR, p53, MERTK, CD44 and OLIG2), we investigated 183 primary and 49 recurrent tumors derived from patients with previously diagnosed dLGG. Of the 49 relapses, nine tumors recurred a second time, and one a third time.

**Results:**

In total, 71.0% of all tumors could be subtyped. Proneural was most dominant in IDH-mut tumors (78.5%), mesenchymal more common among IDH-wt tumors (63.6%). There was a significant difference in survival between classical, proneural and mesenchymal phenotypes in the total cohort (p<0.001), but not after molecular stratification (IDH-mut: p = 0.220, IDH-wt: p = 0.623). Upon recurrence, proneural was retained in 66.7% of the proneural IDH-mut dLGGs (n = 21), whereas IDH-wt tumors (n = 10) mainly retained or gained mesenchymal phenotype. No significant difference in survival was found between IDH-mut gliomas remaining proneural and those shifting to mesenchymal phenotype (p = 0.347).

**Conclusion:**

Subtyping into classical, proneural and mesenchymal phenotypes by five immunohistochemical markers, was possible for the majority of tumors, but protein signatures did not correlate with patient survival in our WHO2021-stratified cohort. At recurrence, IDH-mut tumors mainly retained proneural, while IDH-wt tumors mostly retained or gained mesenchymal signatures. This phenotypic shift, associated with increased aggressiveness in glioblastoma, did not affect survival. Group sizes were, however, too small to draw any firm conclusions.

## Introduction

Adult-type diffuse gliomas are primary tumors of the central nervous system and characterized by infiltrative growth and the development of treatment resistance [1]. The clinical variety within gliomas is large and individual outcome is difficult to predict, especially in gliomas of lower grade [1,2]. Previously, glioblastoma (GBM) as well as lower-grade gliomas comprised both isocitrate dehydrogenase gene mutant (IDH-mut) and IDH *wild-type* (IDH-wt) tumors [3]. In the WHO 2021 classification, however, IDH-mut tumors are separated from IDH-wt tumors [1]. Adult-type diffuse gliomas of lower grade (dLGG) now exclusively comprise IDH-mutated tumors, while IDH-wt is reserved for GBM grade 4 only, also in case of histological lower-grade [1]. Furthermore, IDH-mut astrocytomas with a homozygous deletion of cyclin dependent kinase inhibitor 2A/B (*CDKN2A/B*) have worse prognosis and are therefore upgraded to WHO grade 4 [1,4].

Additional classification systems would be welcomed to explain the differences in outcome within the WHO-defined subclasses. One such example is the Verhaak-classification [5], originally described for GBM, based on large scale mRNA-expression data from The Cancer Genome Atlas. In this classification, four different subclasses of GBM were reported to correlate with clinical outcome: Proneural, Neural, Classical and Mesenchymal. Since then, the Neural subtype has been identified as normal neural lineage contamination, reducing the transcriptomic signatures to classical, proneural and mesenchymal [6,7]. In line with the findings from Verhaak *et al* [5], several studies found that GBM with mesenchymal phenotype carried the worst prognosis, while longer survival was seen for patients with a proneural phenotype, attributed to the association with IDH-mutation [5,8–11]. When removing IDH-mut gliomas from proneural GBMs, no survival benefit could be seen, instead these tumors had even worse survival than other groups [12,13]. Similarly, classification into proneural, classical and mesenchymal subtypes was shown to be relevant also for gliomas of lower grade, although its clinical implications not as thoroughly studied [8,14,15]. In dLGG, proneural was the most dominant and prognostically favorable subgroup compared to other groups [5,14,15].

In later years, the plasticity of the glioma phenome has received increased attention [7,16–18] As such, the transition between phenotypic states–and especially the shift towards a mesenchymal signature–in response to selective pressures occurring between primary diagnosis and recurrence, has emerged as an escape mechanism of the tumor contributing to therapy resistance [7,16,19]. In general, GBMs that have undergone mesenchymal transformation are associated with increased aggressiveness, alterations of the immune microenvironment and multi-therapy resistance [7,19–21].

Despite the advent of molecular profiling becoming an integrated part of brain tumor diagnostics, immunohistochemistry has still an established role as a fast and resource-effective surrogate for large-scale screening of molecular aberrations [22–24]. In a study from 2014, Popova *et al* showed that immunohistochemical analysis using five “key markers”–EGFR, p53, MERTK, CD44 and OLIG2 –was sufficient to subtype gliomas into proneural, classical and mesenchymal phenotype [25]. The clinical implications of this finding were not further examined.

Here, we set out to investigate this issue, using a well-defined clinical cohort of 183 patients with previously diagnosed dLGG. Our aim was to: 1) examine if the proneural, classical and mesenchymal subtyping based on the detection of immunohistochemical markers according to Popova *et al* [25], correlated to patient survival after re-classification of tumors according to the 2021 WHO criteria, and 2) explore if switches in phenotype occurred over time between primary and relapsing tumors 3) and if so, find out whether transition of phenotype at relapse predicted outcome.

## Materials and methods

### Patient cohort and clinical variables

The study cohort included 183 patients, ≥ 18 years of age, with histologically verified WHO grade 2 or 3 diffuse gliomas, operated at Sahlgrenska University Hospital, Gothenburg, Sweden between 2007 and 2016 and included in our institutional dLGG database [26]. A total of 183 primary tumors and 49 recurrent tumor samples were examined. Clinical data was obtained from electronic patient records with end of follow up Jan 1^st^, 2022.

### Molecular classification

The histopathological diagnosis was made in accordance with the WHO criteria valid at the time of surgery, but re-classified according to the 2021 WHO CNS classification [1] using a combination of clinical histology, immunohistochemistry, fluorescent in situ hybridization, Sanger sequencing and DNA-methylation profiling, as previously described [27]. DNA methylation profiling included tumor classification using a DNA methylation-based classifier (MNP, version 11b4, https://www.molecularneuropathology.org/mnp) [28]. *CDKN2A/B* homozygous deletion, *EGFR*-amplification and chr7+/chr10- status was visually assessed from chromosomal copy number variation profiles (CNV) retrieved from the methylation array data. Data on *TERT* promoter mutation status was not available.

**Fig 1:**
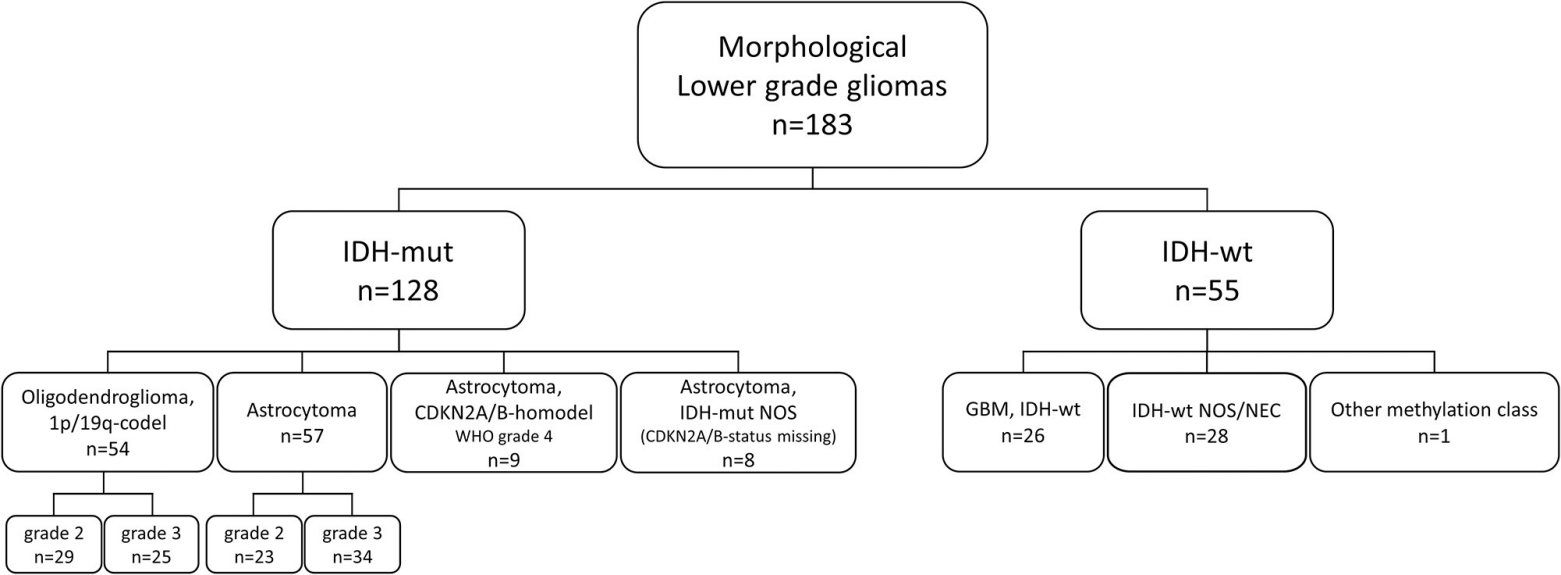
Distribution of the lower-grade gliomas after re-classification according to the WHO classification of 2021.

As shown in Fig 1, IDH-mut tumors (n = 128) were categorized into four subclasses:

1. Oligodendroglioma, IDH-mut and 1p/19q-codeleted, WHO CNS grade 2–3
2. Astrocytoma, IDH-mut, WHO CNS grade 2–3
3. Astrocytoma, IDH-mut, *CDKN2A/B* homozygous deletion, WHO CNS grade 4
4. IDH-mut astrocytoma not otherwise specified (NOS), if *CDKN2A/B* status was missing.

IDH-wt astrocytomas (n = 55) were classified into IDH-wt GBM (n = 26) using methylation-based profiling, if

1. *EGFR*-amp or +chr7/-chr10 was present by visual assessment from CNV profiles, or
2. if the sample was classified as GBM, IDH-wt, by the methylation-based classifier (with a class prediction score ≥ 0.84).

IDH-wt tumors without methylation profile (n = 16), or not meeting any of the above criteria, were grouped and denoted as IDH-wt not otherwise specified/not elsewhere classified (NOS/NEC) (n = 28). One tumor sample was classified as anaplastic pleomorphic xanthoastrocytoma by the methylation-based classifier and therefore excluded from analyzes (denoted as “other methylation class” in Fig 1).

### Tissue handling, tissue microarray construction and immunohistochemical staining

Tissue handling was performed as previously described [29]. In brief, tumor tissue was fixed with 4% formaldehyde, dehydrated and embedded in paraffin wax, followed by a histopathology work up including routine stains, immunohistochemistry and molecular genetic analyses. Tissue microarray (TMA) construction and immunohistochemical staining was performed in collaboration with the Human Protein Atlas consortium [30] (www.proteinatlas.org), at the Tissue profiling site, Uppsala University, Sweden, as previously described [31]. TMAs included two representative tissue cores, 1mm in diameter each, sampled from each formalin-fixed tumor block. For the five protein targets, the following antibodies were used: anti-p53 (M7001, Agilent, 1:750), anti-OLIG2 (HPA003254, Atlas Antibodies AB, 1:1000), anti-CD44 (HPA005785, Atlas Antibodies AB, 1:300), anti-EGFR (HPA018530, Atlas Antibodies AB, 1:100), anti-MERTK (HPA036196, Atlas Antibodies AB, 1:150).

### Immunohistochemical evaluation

**Fig 2:**
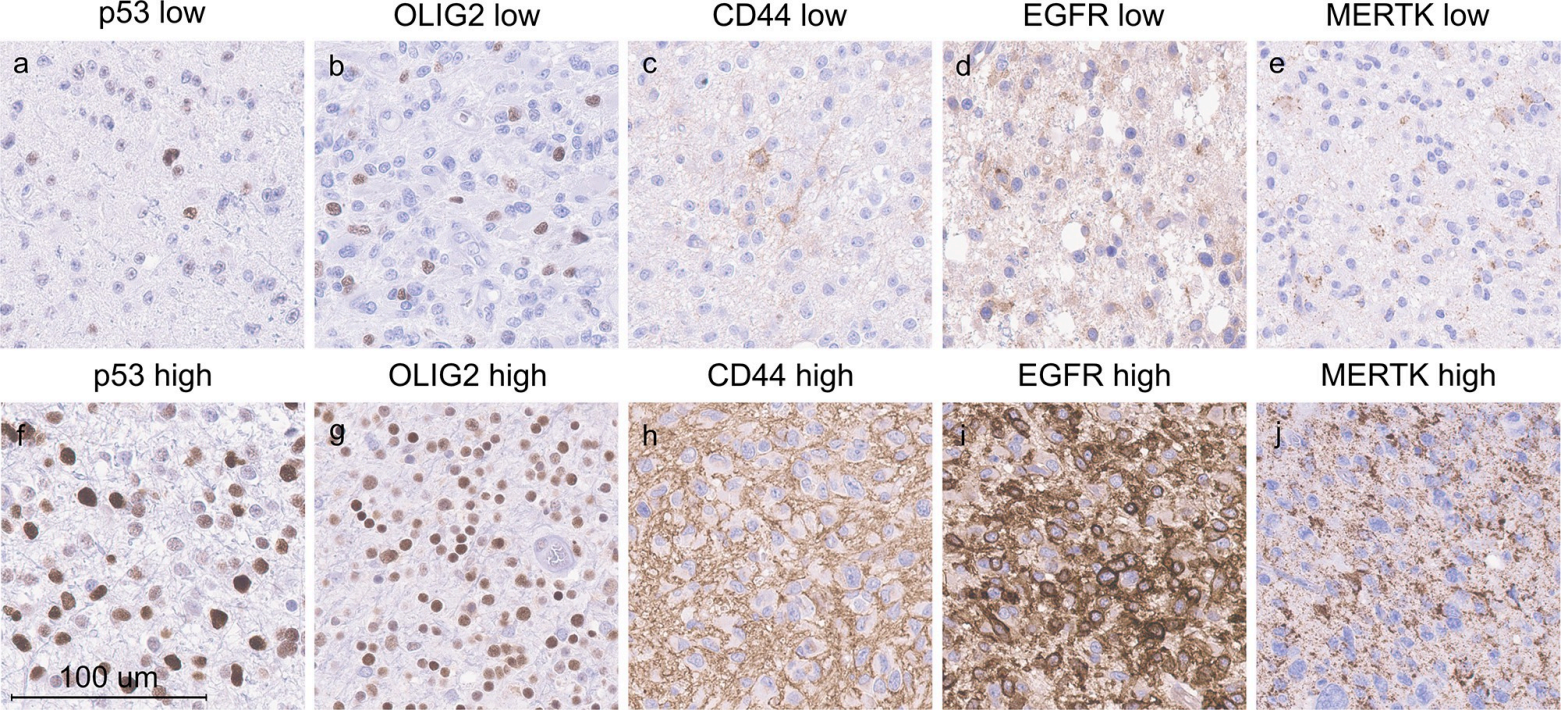
Representative examples of low (a-e) and high (f-j) proportions of immuno-positive cells for the five protein markers used for subtyping.

Evaluation and annotation of the immunohistochemical stainings were performed by two independent evaluators under supervision of a neuropathologist (TOB) and blinded to the clinical status of the subjects. As previously described by Popova *et al [25]*, the proportion of immune-positive cells for each core was assessed and graded on a scale from 0 to 3; Nuclear staining of p53 was scored 0) for absence of nuclear staining, 1) for < 10% positive nuclei, 2) for 10–30% positive nuclei, and 3) for >30% positive nuclei, where scores ≥2 were considered to indicate high presence of p53. Sections stained for OLIG2, CD44, MERTK and EGFR were scored 0) for absence of immunoreactivity, 1) for <10% positivity, 2) for 10–50% of positivity and 3) for >50% positivity. For these proteins, a score of 3 was considered to indicate high expression. Examples of low and high proportions of immuno-positivity can be seen in Fig 2.

### Subtyping

Subtyping of glioma samples into proneural, classical and mesenchymal was performed according to the algorithm presented by Popova and collegues [25], reminiscent of the molecular subtypes defined by Verhaak *et al* [5]. In short, classical subtype included tumors assigned high EGFR and negative p53 expression, mesenchymal subtype included tumors assigned high CD44 and/or high MERTK expression and proneural subtype included tumors assigned high p53 and/or high OLIG2 expression. The algorithm is constructed in a hierarchical fashion, i.e. first selecting cases qualified as classical subtype, followed by mesenchymal, proneural and ultimately categorized into “Other” if none of the former criteria can be applied in this set order. Consequently, as cases are assigned a certain subtype, they will be removed from subsequent steps leaving each case only to be assigned one possible subtype. This subtyping algorithm was performed for primary tumors as well as relapses.

### Statistics

Statistical analyses were performed with IBM SPSS Statistics (Chicago, IL, USA), version 28. Statistical significance level was set to p<0.05. Post-operative survival was defined as the time-period between surgery and the date of death or the end of study (1 Jan 2022) and estimated by Kaplan-Meier method. Differences between groups were compared using log-rank test. Patients alive at the end of study or deceased from other causes (n = 4) (1 prostate cancer, 1 lung cancer, 1 gastric cancer, 1 pancreatic cancer) were censored.

### Ethical approval

This project was approved by the Regional ethical review board in Gothenburg, Sweden (DNR 1067–16). The need for informed consent was waived by the ethical committees. The study was conducted in accordance with the Declaration of Helsinki.

## Results

### Subtyping of primary tumors by immunohistochemistry

Baseline clinical characteristics are presented in Table 1. In the total cohort of 183 patients, 130 tumors (71.0%) could be subtyped into proneural, mesenchymal or classical phenotype by means of the immunohistochemical markers, as proposed by Popova *et al*. A total of 52 tumors (28.4%) did not meet any of the criteria and thus fell into the category denoted as “Other”. One case could not be subtyped due to missing tissue cores in the TMA. One sample subtyped as mesenchymal was classified as anaplastic pleomorphic xanthoastrocytoma and therefore excluded from further analyzes.

**Table 1:** Baseline clinical characteristics in the dLGG study cohort.

	Total cohort	IDH-mut	IDH-wt
Number of patients	183	128	55
Female, n (%)	73 (39.9)	54 (42.2)	19 (34.5)
Age, median (Q1/Q3)	45 (34/57)	40 (32/51)	56 (46/64)
Mainly frontal location, n (%)	97 (53.0)	84 (65.6)	13 (23.6)
Seizure at onset, n (%)	133 (72.7)	97 (75.8)	36 (65.5)
Focal deficit, n (%)	41 (22.4)	20 (15.6)	21 (38.2)
Biopsy only, n (%)	24 (13.1)	6 (4.7)	18 (32.7)

**Fig 3:**
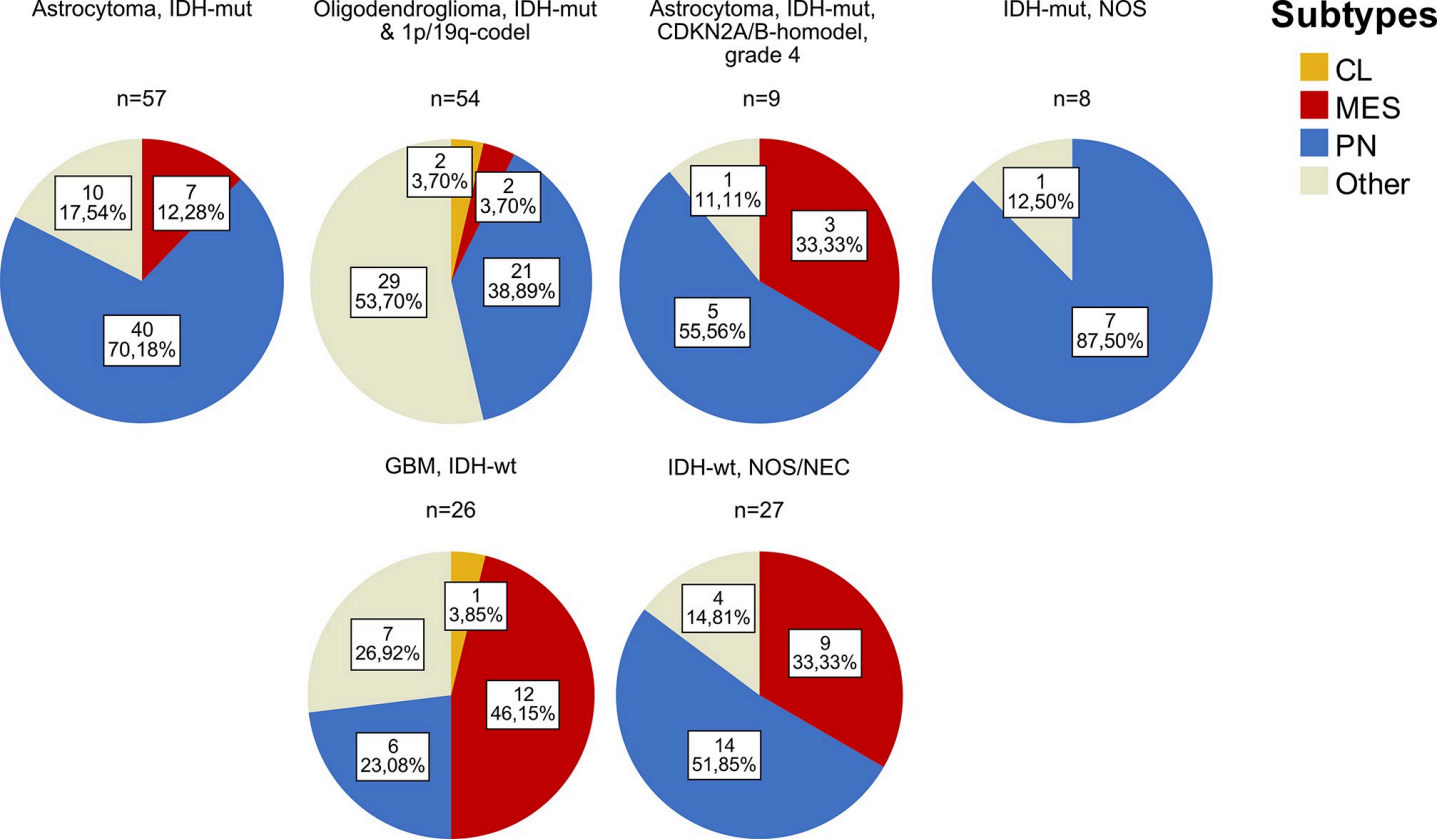
Stratification of the 2021 WHO reclassified primary tumors into proneural (PN), mesenchymal (MES) and classical (CL) subtypes (n = 181). Tumor cases that could not be subtyped are denoted as “Other”.

As visualized in Fig 3, proneural was the predominant phenotype in IDH-mut tumors (57.0%). Proneural dominance was present across all IDH-mut tumor subclasses (70.2%, n = 40 in astrocytomas WHO grade 2–3; 38.9%, n = 21 in oligodendrogliomas WHO grade 2–3; 55.6%, n = 5 in astrocytoma WHO grade 4). Among the oligodendrogliomas, however, more than half of the samples (53.7%) failed to be subtyped and fell into the category of “Other”. The majority of cases assigned as mesenchymal could be found among the group of IDH-wt tumors (63.6%, n = 21). In IDH-wt GBM, mesenchymal was the predominant subtype (46.2%, n = 12).

### Subtyping of relapsed tumors by immunohistochemistry

Of the 183 patients, 49 (26.8%) patients were operated for a recurrent tumor. Of these, 32 cases (65.3%) could be assigned to a subgroup and 15 cases (30.6%) failed specific subtyping and thus fell into “Other”. Two recurrent cases were excluded from immunohistochemical analysis due to missing tissue cores in the TMA.

A detailed overview of subtypes and subtype changes occurring between first and second operation is displayed in S1 Table (IDH-mut tumors) and S2 Table (IDH-wt tumors). None of the primary tumors with classical subtype (n = 3) were found in the relapsed dataset.

**Fig 4:**
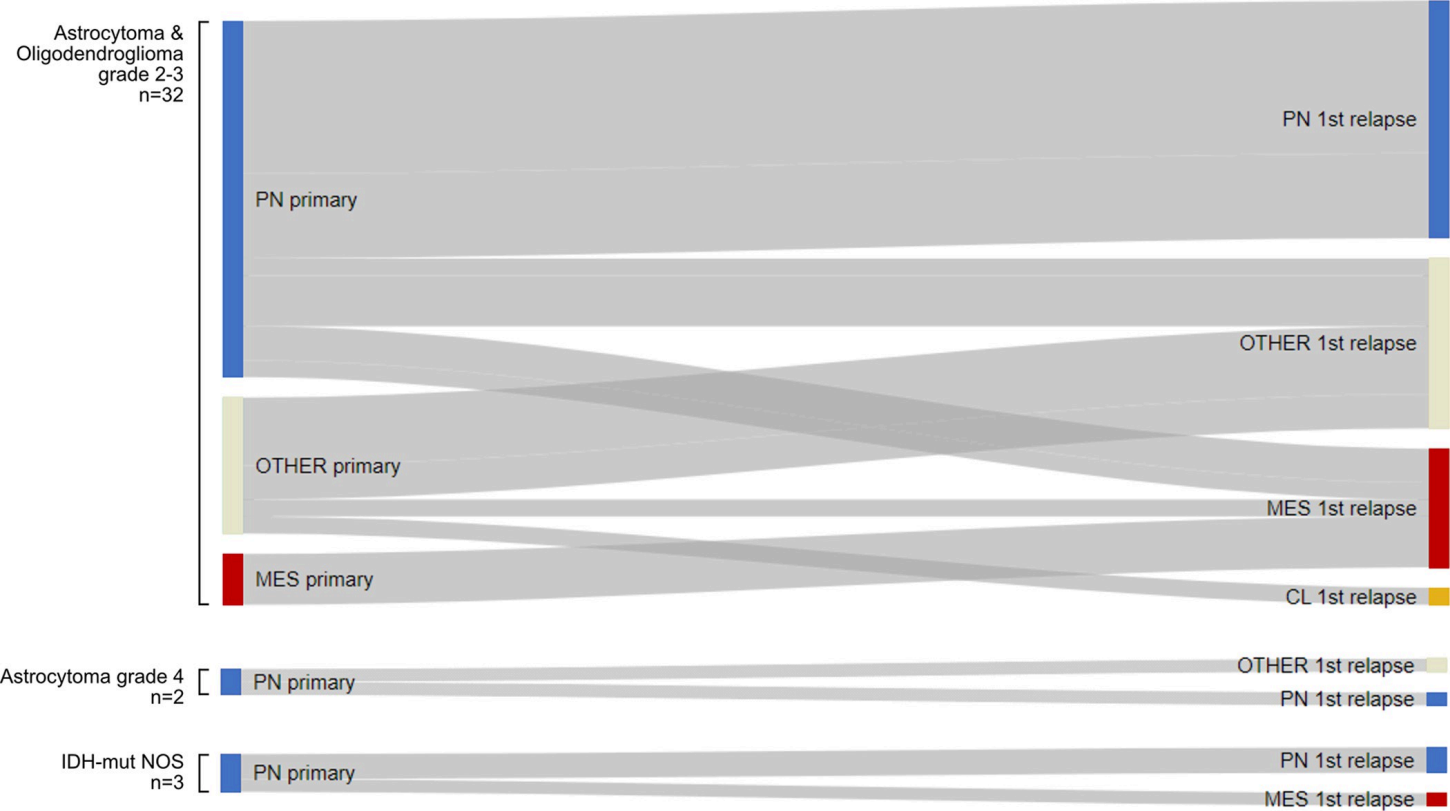
Sankey plot of the primary IDH-mutant (left) and paired 1st relapses (right) subtyped as proneural (PL), mesenchymal (MES), classical (CL) or “Other”.

As illustrated in Fig 4, two thirds of the proneural IDH-mut astrocytomas and oligodendrogliomas of WHO grade 2 and 3 retained proneural phenotype in the recurrent tumor (66.7%, n = 14). Three cases switched from proneural to mesenchymal phenotype (14.3%) and four failed subtyping at relapse. The three cases that were subtyped as mesenchymal in the primary tumor, retained mesenchymal phenotype also in the relapse. In addition, two samples classified as “Other” in the primary tumor gained a specific subtype upon relapse (1 mesenchymal and 1 classical).

All relapsed astrocytoma grade 4 and IDH-mut NOS cases, were of proneural subtype in the primary tumor. The proneural phenotype was retained upon relapse in 3 of 5 cases, one case switched to mesenchymal phenotype and one failed characterization.

Ten primary IDH-wt tumors were operated at relapse; of these, only 6 samples allowed to be subtyped (S2 Table). None of these 6 switched to proneural in the recurrent tumor. Instead, they kept their mesenchymal phenotype (n = 2) or switched to either mesenchymal (n = 2) or classical (n = 1). One sample that failed characterization in the primary tumor could be classified as mesenchymal in recurrent tumor.

Nine tumors in the total cohort of 183 gliomas relapsed a second time, of which one relapsed a third time (Table 2). The latter, initially denoted as “Other” in the primary tumor, showed mesenchymal phenotype in all three relapses. This patient was diagnosed with an IDH-mut astrocytoma grade 2 and survived four years after primary surgery.

**Table 2:** Subtype development in the nine (n = 9) patients that relapsed a second time in the dLGG cohort. Only one (n = 1) patient relapsed a third time.

Subtype in primary tumor	Subtype in 1st relapse	Subtype in 2nd relapse	Subtype in 3rd relapse	WHO2021 class in primary tumor
**PN**	PN	PN		Oligodendroglioma, IDH-mut & 1p/19q-codel, grade 2
**PN**	PN	PN		IDH-mut Astrocytoma, NOS (morphologically grade 2)
**PN**	MES	PN		IDH-mut Astrocytoma, NOS (morphologically grade 2)
**MES**	MES	MES		Astrocytoma, IDH-mut, grade 2
**MES**	MES	MES		GBM, IDH-wt
**Other**	Other	MES		Oligodendroglioma, IDH-mut & 1p/19q-codel, grade 2
**Other**	MES	MES	MES	Astrocytoma, IDH-mut, grade 2
**Other**	MES	MES		GBM, IDH-wt
**Other**	Classical	PN		Oligodendroglioma, IDH-mut & 1p/19q-codel, grade 2

### Correlation with survival

**Fig 5:**
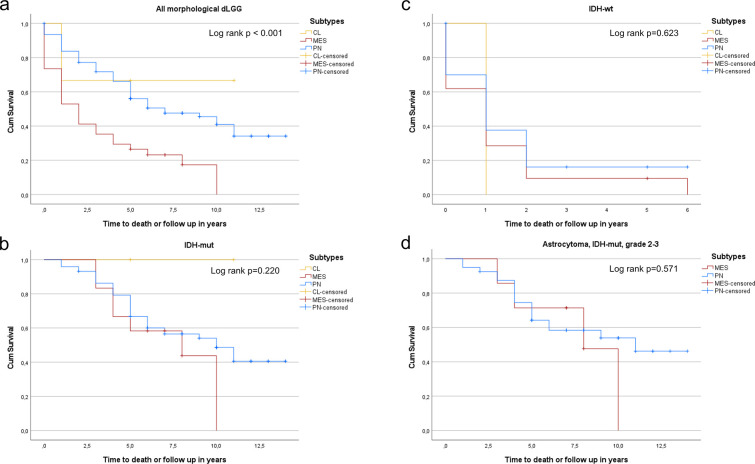
**a.** Kaplan-Meier curves of PN (n = 93), MES (n = 33) and CL (n = 3) tumors in the total cohort of low-grade gliomas (PN: 7.0 years, 95% CI 3.45–10.55, MES: 2.0 years, 95% CI 0.98–3.02, and CL: Median survival not reached). **b.** Kaplan-Meier curves of PN (n = 73), MES (n = 12) and CL (n = 2) subtypes in IDH-mut gliomas (PN: 10.0 years, 95% CI 6.70–13.30, MES: 8.0 years, 95% CI 1.33–14.67, and CL: All cases censored). **c.** Kaplan-Meier curves of PN (n = 20), MES (n = 21) and CL (n = 1) subtypes in IDH-wt gliomas (PN: 1.0 years, 95% CI 0.32–1.68, MES: 1.0 years, 95% CI 0.42–1.58, and CL: 1 year, 95% CI could not be calculated). **d.** Kaplan-Meier curves of PN (n = 40) and MES (n = 7) subtypes in IDH-mut astrocytomas of grade 2–3 (PN: 11.0 years, 95% CI could not be calculated, MES: 8,0 years, 95% CI 4.30–11.71). There were no IDH-mut astrocytomas of CL phenotype.

There was a significant difference in survival between subtypes in the total cohort (Fig 5a, log rank p<0.001), but not when analyzing IDH-mut or IDH-wt gliomas separately (Fig 5B and 5C, log rank p = 0.220 and log rank p = 0.623). Tumors denoted as “Other” were excluded from survival analyzes. No significant difference in survival was seen between proneural (n = 40) and mesenchymal (n = 7) tumors analyzing IDH-mut astrocytomas of lower grade (Fig 5D; log rank p = 0,571). Log-rank testing between subgroups in oligodendrogliomas was not considered meaningful, due to the small subgroups of mesenchymal (n = 2) and classical (n = 2), alongside with a large unclassified group of "Other" (n = 29). A summary of results, showing median estimated survival for patients with tumors classified by the WHO 2021 criteria, and grouped into the defined subclasses, is shown in S3–S5 Tables.

**Fig 6:**
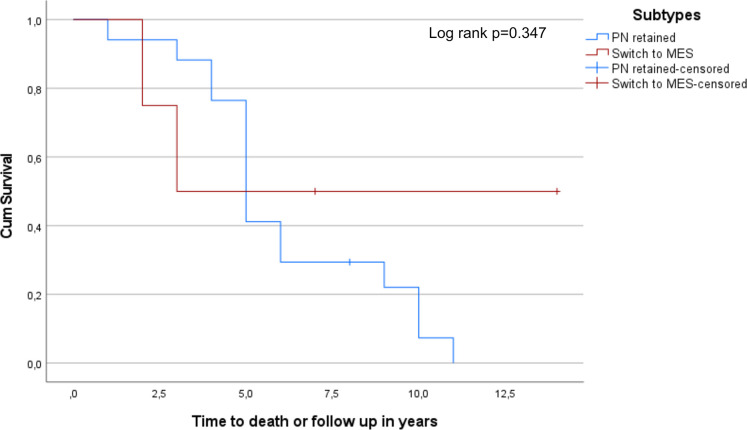
Kaplan-Meier plot of the 17 IDH-mut samples that retained PN phenotype upon relapse compared to the 4 IDH-mut samples that switched from PN phenotype to MES (PN retained: 5.0 years, 95% CI 4.34–5.66, Switch to MES: 3.0 years, 95% CI could not be calculated).

In a final step, we examined if there was a difference in survival between patients with IDH-mut tumors that retained proneural subtype upon relapse (n = 17) and those that switched from proneural to mesenchymal subtype (n = 4). As shown in Fig 6, sample sizes were small and no significant difference was found (log rank p = 0.347).

### Discussion

We found an association between tumor subtypes (proneural, classical, mesenchymal) and outcome in a clinical cohort previously classified as lower-grade gliomas. However, after molecular stratification of tumors according to the WHO 2021 criteria the statistical significance between phenotypes and survival was lost. Although a proneural to mesenchymal transition was the most consistent change between primary and recurrent tumor, this phenotypic shift provided no apparent prognostic information in our cohort. Small sample sizes, however, did not allow to draw any conclusions on whether this negative finding is due to lack of statistical power or reflects a true biological difference from glioblastomas.

### Subtypes in primary tumor and association with survival

Previous studies of molecular signatures in lower-grade glioma have mainly been based on a mixture of IDH-mut and *IDH*-wt tumors with different malignancy grades, which makes comparisons difficult. The present study illustrates how the above-described subtyping scheme can be applied in a cohort of more homogenously defined dLGGs, classified according to the recent WHO classification. As expected, the IDH-mut dLGGs were mainly of proneural subtype [5,8,14], while IDH-wt gliomas with molecular features of GBM more often were mesenchymal. In line with our findings, previous studies showed that the classical signature is frequent in GBMs, but less common in grade 2 and 3 tumors [14,15]. The proneural dominance in IDH-mut tumors was a confounding factor, explaining the significantly longer survival correlated with this subtype in the total cohort. No such difference in outcome between phenotypes was found within the more uniform cohort of IDH-mut tumors, nor within the group of astrocytomas of WHO grade 2–3 or IDH-wt tumors. Thus, the prognostic information provided by this subtyping and the clinical usefulness of this method for currently relevant molecular subclasses are not obvious.

### Subtypes in relapsing tumors

An additional aim of this study was to apply the immunohistochemistry-based subtyping in relapsing tumors and investigate if subtype switches occurred over time in progressive disease. To our knowledge, such switches in protein profiles between first and second surgery, have not been described in dLGG classified by the WHO2021 criteria. The majority of the primary IDH-mut tumors kept the proneural phenotype upon recurrence, which is in line with a recent report by Varn et al [32], using molecularly verified data. In their study, 78% of the IDH-mut tumors retained the proneural subtype at recurrence. In the same study, 49% of patients with IDH-wt tumors switched subtype at relapse, where a transition to mesenchymal was the most common. In our cohort, recurrent IDH-wt tumors mainly kept their mesenchymal phenotype or switched to mesenchymal, while none transformed to proneural phenotype.

Accumulating evidence show that GBMs undergo mesenchymal transformation due to changes in microenvironment and inherent physical structure, highlighting the importance of investigating the underlying mechanisms and associated–seemingly dynamic [16]–therapeutic targets [6,7]. In IDH-mut gliomas, however, the mesenchymal switch is seemingly not the major contributor to tumor progression and treatment resistance.

### Strengths and limitations

A limitation is the skewness of the cohort, with only few samples with mesenchymal and classical phenotypes, and the subsequent lack of statistical power. This problem must still be accounted when studying larger cohorts, since the majority of IDH-mut dLGG are of proneural phenotype. Another methodological limitation concerns the subjectivity of immunohistochemistry in evaluation and inter-observer variability. This might explain noncongruence between findings, e.g., the large group of “Other” (28%) that failed to meet the subtyping criteria. This relatively large unclassified group warrants caution in interpreting the data. In the original study by Popova et al [25], the percentage low-grade gliomas denoted as “Other” was only 15%. However, this study followed the WHO 2007 criteria, including grade 2 gliomas only and lacking diagnostic molecular markers. A more general limitation of the TMA method is the small amount of tissue being available for immunohistochemical analysis, with a risk for not capturing the full bulk of phenotypic variance [33]. The main strength of the study is the long follow-up time of patients in combination with the comprehensive clinical data.

### Summary and future perspectives

Immunohistochemistry offers a simple diagnostic detection method, also in settings with less resources. Subtyping according to the scheme described in this report was possible for the majority of tumors, prognostic in the lower-grade group as a whole, but not in the separate entities defined by the WHO2021 classification. However, the method gives a snapshot of the biological behavior of adult-type lower-grade diffuse gliomas, in primary as well as relapsing tumors. Most IDH-mut tumors with proneural subtype retained proneural phenotype at relapse, while few gained mesenchymal phenotype. The prognostically unfavorable group of IDH-wt tumors was indeed associated with a more aggressively mesenchymal-linked phenotype and likely to retain or gain this phenotype at recurrence [6,19,32]. Whether mesenchymal transition over time, as well as the intricate relationship with microenvironment, treatment sensitivity and treatment resistance, has a similar role in IDH-mut gliomas will need to be clarified in future studies.

## Supporting information

S1 Table Subtypes in primary IDH-mutated glioma samples compared to subtype in the corresponding relapsed sample. CL = Classical, MES = Mesenchymal, PN = Proneural.
(DOCX)

S2 Table Subtypes in primary, morphologically low grade, IDH-wt glioma samples compared to subtype in the corresponding relapsed sample. CL = Classical, MES = Mesenchymal, PN = Proneural.
(DOCX)

S3 Table Distribution of primary tumor subtypes and median estimated survival in dLGGs, IDH-mut astrocytomas of WHO CNS grade 2–3, and IDH-mut and 1p/19q-codeleted oligodendrogliomas WHO CNS grade 2–3. CL = Classical, MES = Mesenchymal, PN = Proneural.
(DOCX)

S4 Table Distribution of primary tumor subtypes and median estimated survival in IDH-mut and CDKN2A/B-homodeleted astrocytomas of WHO CNS grade 4. CL = Classical, MES = Mesenchymal, PN = Proneural.
(DOCX)

S5 Table Distribution of primary tumor subtypes and median estimated survival in GBM, IDH-wt, morphologically low grade. CL = Classical, MES = Mesenchymal, PN = Proneural.
(DOCX)
