# Supplementary material for: The clinical value of proneural, classical and mesenchymal protein signatures in WHO 2021 adult-type diffuse lower-grade gliomas
Source: PLoS One. 2023 May 16;18(5):e0285732. doi: 10.1371/journal.pone.0285732 (PMC10187920; doi:10.1371/journal.pone.0285732)
Supplement: S1 Table — CL = Classical, MES = Mesenchymal, PN = Proneural. (DOCX) [file pone.0285732.s001.docx]

**S1 Table.** **Subtypes in** **primary IDH-mutated glioma samples compared to subtype in the corresponding relapsed sample.** CL=Classical, MES=Mesenchymal, PN=Proneural.

|  | Subtype in first relapse | | | | | | | | | | | | | | | |
| --- | --- | --- | --- | --- | --- | --- | --- | --- | --- | --- | --- | --- | --- | --- | --- | --- |
|  | Astrocytoma, IDH-mut, grade2-3  (n=20) | | | | Oligodendroglioma, IDH-mut, grade2-3  (n=12) | | | | Astrocytoma, IDH-mut, grade 4  (n=2) | | | | IDH-mut NOS  (n=3) | | | |
| Subtype in primary tumor | CL, n | MES, n | PN, n | OTHER, n | CL, n | MES, n | PN, n | OTHER, n | CL, n | MES, n | PN, n | OTHER, n | CL, n | MES, n | PN, n | OTHER, n |
| CL | n/a | n/a | n/a | n/a | n/a | n/a | n/a | n/a | n/a | n/a | n/a | n/a | n/a | n/a | n/a | n/a |
| MES | 0 | 3 | 0 | 0 | 0 | 0 | 0 | 0 | 0 | 0 | 0 | 0 | 0 | 0 | 0 | 0 |
| PN | 0 | 2 | 9 | 1 | 0 | 1 | 5 | 3 | 0 | 0 | 1 | 1 | 0 | 1 | 2 | 0 |
| Other | 0 | 1 | 0 | 4 | 1 | 0 | 0 | 2 | 0 | 0 | 0 | 0 | 0 | 0 | 0 | 0 |
| Total | 0 | 6 | 9 | 5 | 1 | 1 | 5 | 5 | 0 | 0 | 1 | 1 | 0 | 1 | 2 | 0 |
