# Supplementary material for: The clinical value of proneural, classical and mesenchymal protein signatures in WHO 2021 adult-type diffuse lower-grade gliomas
Source: PLoS One. 2023 May 16;18(5):e0285732. doi: 10.1371/journal.pone.0285732 (PMC10187920; doi:10.1371/journal.pone.0285732)
Supplement: S3 Table — CL = Classical, MES = Mesenchymal, PN = Proneural. (DOCX) [file pone.0285732.s003.docx]

**S3 Table. Distribution of primary tumor subtypes and median estimated survival in dLGGs, IDH-mut astrocytomas of WHO CNS grade 2-3, and IDH-mut and 1p/19q-codeleted oligodendrogliomas WHO CNS grade 2-3.** CL=Classical, MES=Mesenchymal, PN=Proneural.

| Subtype in primary tumor | dLGG, IDH-mut | | | Astrocytoma, IDH-mut, grade 2-3 | | | Oligodendroglioma, 1p/19q-codel, grade 2-3 | | |
| --- | --- | --- | --- | --- | --- | --- | --- | --- | --- |
|  | n (%) | Median survival in years  (95% CI) | No of deceased | n (%) | Median survival in years  (95% CI) | No of deceased | n (%) | Median survival in years  (95% CI) | No of deceased |
| CL | 2 (1,8) | Not reached | 0 | 0 (0) | n/a | 0 | 2 (3,8) | Not reached | 0 |
| MES | 9 (8,2) | 10,0 * | 5 | 7 (12,3) | 8,0 (4,291-11,709) | 4 | 2 (3,8) | 10,0* | 1 |
| PN | 61 (55,5) | 11* | 26 | 40 (70,2) | 11,0* | 18 | 21 (39,6) | 11,0* | 8 |
| Other | 38 (34,5) | Not reached | 8 | 10 (17,5) | 8,0 (5,608-10,392) | 5 | 28** (52,8) | Not reached | 3 |
| Total | 110 (100,0) | Not reached | 39 | 57 (100,0) | 10,0 (6,553-13,447) | 27 | 53 (100,0) | Not reached | 12 |

* CI could not be calculated

** One case was missing survival data.
