# Supplementary material for: The clinical value of proneural, classical and mesenchymal protein signatures in WHO 2021 adult-type diffuse lower-grade gliomas
Source: PLoS One. 2023 May 16;18(5):e0285732. doi: 10.1371/journal.pone.0285732 (PMC10187920; doi:10.1371/journal.pone.0285732)
Supplement: S4 Table — CL = Classical, MES = Mesenchymal, PN = Proneural. (DOCX) [file pone.0285732.s004.docx]

**S4 Table.** **Distribution of primary tumor subtypes and median estimated survival in IDH-mut and CDKN2A/B-homodeleted astrocytomas of WHO CNS grade 4.** CL=Classical, MES=Mesenchymal, PN=Proneural.

| Subtype in primary tumor | Astrocytoma, IDH-mut, CDKN2A/B-homodel, grade 4 | | |
| --- | --- | --- | --- |
|  | n (%) | Median survival in years  (95% CI) | No of deceased |
| CL | 0 | n/a | 0 |
| MES | 3 | 4,0 (2,400-5,600) | 3 |
| PN | 5 | 5,0 (0,706-9,292) | 3 |
| Other | 1 | 5,0* | 1 |
| Total | 9 | 5,0 (4,185-5,815) | 7 |

* CI could not be calculated
