# Supplementary material for: The clinical value of proneural, classical and mesenchymal protein signatures in WHO 2021 adult-type diffuse lower-grade gliomas
Source: PLoS One. 2023 May 16;18(5):e0285732. doi: 10.1371/journal.pone.0285732 (PMC10187920; doi:10.1371/journal.pone.0285732)
Supplement: S5 Table — CL = Classical, MES = Mesenchymal, PN = Proneural. (DOCX) [file pone.0285732.s005.docx]

**S5 Table.** Distribution of primary tumor subtypes and median estimated survival in GBM, IDH-wt, morphologically low grade.

| Subtype in primary tumor | GBM, IDH-wt | | |
| --- | --- | --- | --- |
|  | n (%) | Median survival in years  (95% CI) | No of deceased |
| CL | 1 (3,8) | 1* | 1 |
| MES | 12 (46,2) | 1 (0,510-1,490) | 12 |
| PN | 6 (23,1) | 1 (0,404-1,596) | 6 |
| Other | 7 (26,9) | 1 * | 7 |
| Total | 26 (100,00) | 1 (0,719-1,281) | 26 |

* CI could not be calculated
